# Substrate Flexibility and Metal Deposition Method Effects on Piezoelectric-Enhanced SERS in Metal–ZnO Nanorod Nanocomposites

**DOI:** 10.3390/ma18143299

**Published:** 2025-07-13

**Authors:** Nguyen Thi Quynh Nhu, Le Tran Thanh Thi, Le Vu Tuan Hung, Vincent K. S. Hsiao

**Affiliations:** 1 Department of Applied Materials and Optoelectronic Engineering, National Chi Nan University, Nantou 54561, Taiwan; s108328502@mail1.ncnu.edu.tw (N.T.Q.N.); s108328503@mail1.ncnu.edu.tw (L.T.T.T.); 2 Department of Applied Physics, Faculty of Physics and Engineering Physics, University of Science, Ho Chi Minh City 748000, Vietnam

**Keywords:** Surface-Enhanced Raman Scattering (SERS), piezoelectric enhancement, ZnO nanorods, flexible substrates, laser-induced photolysis, metal deposition method, substrate flexibility

## Abstract

This study investigates the effects of substrate flexibility and metal deposition methods on piezoelectric-enhanced Surface-Enhanced Raman Scattering (SERS) in metal-deposited ZnO nanorod (NR) nanocomposites (NCPs). ZnO NRs were grown on both rigid (ITO–glass) and flexible (ITO-PET) substrates, followed by gold (Au) deposition by pulsed-laser-induced photolysis (PLIP) or silver (Ag) deposition by thermal evaporation. Structural analysis revealed that ZnO NRs on flexible substrates exhibited smaller diameters (60–80 nm vs. 80–100 nm on glass), a higher density, and diverse orientations that enhanced piezoelectric responsiveness. Optical characterization showed distinct localized surface plasmon resonance (LSPR) peaks at 420 nm for Ag and 525 nm for Au systems. SERS measurements demonstrated that Ag-ZnO NCPs achieved superior detection limits (10^−9^ M R6G) with enhancement factors of 10^8^–10^9^, while Au-ZnO NCPs reached 10^−8^ M detection limits. Mechanical bending of flexible substrates induced dramatic signal enhancement (50–100-fold for Au-ZnO/PET and 2–3-fold for Ag-ZnO/PET), directly confirming piezoelectric enhancement mechanisms. This work establishes quantitative structure–property relationships in piezoelectric-enhanced SERS and provides design principles for high-performance flexible sensors.

## 1. Introduction

Surface-Enhanced Raman Scattering (SERS) has emerged as a powerful analytical technique with exceptional sensitivity for molecular detection [1]. Since its first observation on roughened silver electrodes in the 1970s [2], SERS has evolved into a sophisticated method capable of single-molecule detection [3]. The technique leverages electromagnetic field enhancement generated by localized surface plasmon resonance (LSPR) at the surface of noble metal nanostructures, significantly amplifying Raman signals and enabling ultra-sensitive structural analysis of trace analytes [4]. SERS offers distinct advantages, including non-destructive analysis, high sensitivity, and molecular specificity, making it widely applicable in biosensing, materials science, environmental monitoring, and chemical analysis [5]. The enhancement mechanisms in SERS primarily involve electromagnetic and chemical contributions. Electromagnetic enhancement originates from LSPR excitation, which concentrates electromagnetic fields in gaps, crevices, or sharp features of noble metal nanostructures, creating “hot spots” with theoretical enhancement factors of 10^10^ to 10^11^ [6]. Chemical enhancement, involving charge transfer processes between metals and adsorbed molecules, contributes additional enhancement factors of approximately 10^3^ [7]. Collectively, optimized SERS substrates can achieve total enhancement factors of 10^10^ to 10^11^, enabling detection of extremely dilute samples and detailed molecular structural analysis.

Gold (Au) and silver (Ag) represent the most used SERS-active metals, exhibiting significant differences in surface plasmon resonance characteristics, biocompatibility, and chemical stability [8]. Ag nanoparticles generally demonstrate stronger plasmon resonance in the visible range (390–700 nm), while Au nanoparticles exhibit resonance peaks at longer wavelengths (520–580 nm). This distinction directly affects their SERS enhancement capabilities at different excitation wavelengths. Under identical conditions, Ag nanoparticles typically provide enhancement factors 10–100 times higher than Au nanoparticles, making Ag preferable for ultra-high-sensitivity detection [9,10,11,12]. However, Au nanoparticles offer superior frequency stability and broader resonance bands, providing relatively stable enhancement across wider wavelength ranges. Additionally, Au nanoparticles’ plasmonic properties can be more precisely controlled by adjusting their shape and size, facilitating application-specific optimization [13]. Au nanoparticles significantly outperform silver in chemical stability. Ag readily oxidizes in air or reacts with sulfur- or chlorine-containing compounds, forming surface oxide layers or sulfides that substantially reduce SERS activity. In contrast, Au exhibits excellent chemical stability, maintaining its plasmonic properties across various environmental conditions—a crucial factor for application stability and reliability. Au nanoparticles also demonstrate superior biocompatibility, with lower toxicity and reduced immunogenicity, making them more suitable for biological sample detection and in vivo applications. Despite these limitations, Ag nanoparticles remain highly attractive for SERS applications due to their superior enhancement factors and cost-effectiveness, making them particularly valuable for high-sensitivity analytical applications where long-term stability is not a primary concern [14]. Metal deposition methods significantly influence nanoparticle characteristics and subsequent SERS performance [15,16]. Pulsed-laser-induced photolysis (PLIP) offers a novel approach for metal nanoparticle preparation, enabling precise control over nanoparticle size and distribution with remarkably short processing times [17,18,19,20]. In this method, sol–gel-prepared ZnO nanorods (NRs) are immersed in a solution containing gold chloride (HAuCl_4_) and hydrogen peroxide (H_2_O_2_) and are then exposed to pulsed-laser irradiation [17]. Within just 30 min, H_2_O_2_ reduces Au ions to form Au nanoparticles without requiring further treatment. This approach allows precise control of Au nanoparticle formation, ensuring uniform distributions on ZnO nanorod surfaces and creating ideal “hot spot” regions for enhanced SERS signals. In contrast, conventional thermal evaporation deposits metal films through vacuum evaporation of source materials onto substrate surfaces. While widely applied, this method has limitations in nanoscale precision control. Thermal evaporation typically requires additional heat treatment or annealing processes to transform continuous metal films into nanoparticle structures, a time-consuming process with challenges in controlling size uniformity and distribution density. Furthermore, high-temperature processing may adversely affect ZnO nanorod structure and piezoelectric properties, potentially diminishing SERS performance.

ZnO, a II-VI semiconductor with a wide bandgap and stable wurtzite structure, has attracted significant attention as a promising SERS substrate material [21,22,23,24,25]. ZnO exhibits exceptional properties, including low toxicity, good biocompatibility, biodegradability, and remarkable piezoelectric characteristics [26]. Unlike noble metals that primarily rely on electromagnetic enhancement, ZnO nanostructures can generate chemical enhancement through interfacial charge transfer processes [21]. When combined with noble metal nanoparticles to form hybrid structures, ZnO can synergistically incorporate both electromagnetic enhancement from metals and interfacial charge transfer processes, further amplifying SERS signals [27]. For instance, in Ag-ZnO hybrid structures [28,29,30,31,32,33,34,35,36,37,38,39,40,41,42,43], electrons transfer from silver (with a higher Fermi level) to ZnO, accumulating near the heterojunction and inducing electromagnetic fields that couple with incident fields to produce significantly enhanced local fields around the heterojunction. The piezoelectric effect—the generation of electric fields in certain materials under mechanical stress—can significantly enhance SERS signals [44,45,46,47,48]. In 2006, Dr. Z. L. Wang’s group introduced the concept of piezotronics and comprehensively investigated ZnO’s piezoelectric capabilities [49]. They explained that mechanical stress application induces redistribution of electric dipoles within ZnO crystals due to structural asymmetry, separating positive and negative charge centers and inducing surface charges. In the absence of stress relaxation, these polarization charges accumulate at the material surface, creating piezoelectric fields within nanowires. His Group also demonstrated that bent ZnO nanowires generate positive and negative charges on tensile and compressive sides, respectively, resulting in internal piezoelectric fields [50]. These polarization charges can electrostatically attract nearby small molecules, concentrating them from dilute solutions onto the material surface. This significantly increases molecular coverage, enhancing sensor sensitivity.

Recent studies have demonstrated that mechanical stress applied to bent ZnO microwires/nanowires generates piezoelectric fields that enhance SERS signals [48]. Deng et al. [48] fabricated ZnO microwires using hydrothermal decomposition, bent them using a 1 μm tungsten probe to apply mechanical stress, and fixed the microwire ends with polymethyl methacrylate (PMMA). Silver nanoparticles were then deposited on the ZnO microwire surface through magnetron sputtering, creating a piezoelectric-enhanced ZnO/Ag microcavity SERS substrate. Raman spectroscopy measurements confirmed peak shifts in the E2 high mode at different positions along the ZnO microwire, indicating varying strain levels. The greatest strain occurred near the microwire’s bending center, where the electric field was strongest, attracting more probe molecules and generating the most intense SERS signals. Signal intensity gradually decreased as measurement positions moved from the center toward the ends, confirming the critical role of piezoelectric fields in SERS enhancement. Substrate selection critically influences SERS substrate performance, particularly in piezoelectric-enhanced SERS systems where mechanical properties directly affect piezoelectric effect generation and utilization efficiency. Traditional SERS substrates often employ rigid materials like glass or silicon, offering good chemical stability and optical transparency for spectroscopic measurements. However, these rigid substrates have limitations when applying piezoelectric effects, as they cannot generate sufficient deformation to activate piezoelectric responses in loaded materials. In contrast, flexible substrates such as PVDF can easily undergo bending, twisting, or stretching during application, generating more pronounced strain distributions in piezoelectric materials and further enhancing piezoelectric effects on SERS signals [45,47].

In this study, we investigate the synergistic effects of substrate flexibility and metal deposition methods on the SERS performance of ZnO nanorod composites, with particular focus on piezoelectric enhancement mechanisms. We systematically compare ZnO NRs grown on rigid (ITO–glass) and flexible (ITO-PET) substrates, followed by gold deposition via PLIP and silver deposition via thermal evaporation. This approach allows us to elucidate the fundamental relationships between substrate properties, metal–semiconductor interfaces, and SERS enhancement mechanisms. By analyzing structural, optical, and spectroscopic properties of these composites, we demonstrate that substrate flexibility dramatically impacts SERS performance through piezoelectric effects. Most significantly, we provide direct evidence of piezoelectric enhancement by comparing SERS signals before and after mechanical deformation of flexible substrates. Our findings reveal that while thermally evaporated Ag-coated ZnO nanocomposites (NCPs) exhibit superior intrinsic SERS activity with detection limits down to 10^−9^ M for R6G, PLIP-fabricated Au coated-ZnO nanostructures demonstrate a remarkably stronger piezoelectric response, with signal enhancement factors of 50–100 upon substrate bending. These insights establish new design principles for high-performance flexible SERS sensors and open promising avenues for applications in wearable biosensing, point-of-care diagnostics, and environmental monitoring, where real-time, ultra-sensitive, and selective detection is required.

## 2. Materials and Methods

### 2.1. Reagents and Materials

The main reagents used in this study included zinc acetate dihydrate (Zn(CH_3_COO)_2_·2H_2_O, 99.5%), monoethanolamine (HOCH_2_CH_2_NH_2_, 99%), 2-methoxyethanol (CH_3_OCH_2_CH_2_OH, 99.5%), zinc nitrate hexahydrate (Zn(NO_3_)_2_·6H_2_O, 98%), hexamethylenetetramine (C_6_H_12_N_4_, 99%), chloroauric acid (HAuCl_4_·3H_2_O, 99.9%), hydrogen peroxide (H_2_O_2_, 30%), and Rhodamine 6G (R6G, 95%). Substrate materials included ITO-coated glass substrates (surface resistivity: 10 Ω/sq) and ITO-coated PET flexible substrates (surface resistivity: 60 Ω/sq). All chemical reagents (Sigma-Aldrich, Merck Ltd., Taipei, Taiwan) were of analytical grade purity and used without further purification.

### 2.2. Preparation of ZnO Nanorods

ZnO seed layers were prepared using the sol–gel method [51]. First, a precursor solution was formulated by dissolving 0.75 M zinc acetate dihydrate in 15 mL of a mixed solvent, which consisted of equal volumes of monoethanolamine and 2-methoxyethanol. The solution was allowed to stabilize at room temperature for 24 h, with periodic stirring to ensure complete dissolution and homogeneous mixing. The seed layers were deposited by a dip-coating method. Cleaned ITO–glass or ITO-PET substrates were immersed in the sol solution for 10 min and then withdrawn at a rate of 3 cm/min. This process was repeated 3–5 times to obtain seed layers of varying thicknesses. After each coating, the substrate was dried at room temperature for 5 min, followed by annealing on a hot plate. For ITO–glass substrates, the annealing temperature was set at 100–250 °C; for ITO-PET substrates, considering the thermal stability of PET, the annealing temperature was controlled at 150 °C. Due to the flexible nature of PET, slight substrate deformation during thermal processing was unavoidable. The annealing time for all samples was 30 min. The growth of ZnO NRs was prepared by immersing the samples in a chemical bath containing a solution of zinc nitrate hexahydrate (Zn(NO_3_)_2_ · 6H_2_O) and methenamine (C_6_H_12_N_4_) at the same 0.1 M concentration at 85 °C for 2 h.

### 2.3. Deposition of Au Nanoparticles (Pulsed-Laser-Induced Photolysis)

Au nanoparticles were deposited using the pulsed-laser-induced photolysis (PLIP) method [17]. ZnO nanorod-grown substrates were placed in a glass vial, and 5 mL of 0.33 mM HAuCl_4_ solution and 2 mL of 10 mM H_2_O_2_ aqueous solution were added. The mixed solution was then irradiated using a Nd:YAG nanosecond pulsed laser (wavelength: 532 nm, pulse energy: 47 mJ, repetition rate: 5 Hz, pulse duration: 6–7 ns). The laser beam was directed to the center of the solution to ensure uniform light exposure of the sample. The irradiation time was set to 10 min, during which the solution color gradually changed from yellow to red, indicating the formation of Au nanoparticles. After irradiation, the samples were kept immersed in the solution for 30 min to optimize the amount of Au nanoparticle deposition. Finally, the samples were thoroughly rinsed with deionized water and dried with nitrogen gas.

### 2.4. Deposition of Ag Thin Films (Thermal Evaporation)

Ag thin films were deposited using the thermal evaporation method. A home-made thermal evaporation coating system was used, with high-purity Ag metal (99.99%) placed in a tungsten boat as the evaporation source material. The ZnO nanorod samples were fixed on a substrate holder approximately 20 cm away from the evaporation source. The chamber was first evacuated to a base pressure of approximately 5 × 10^−5^ Torr, then the tungsten boat was gradually heated to initiate Ag evaporation. The evaporation rate was controlled at 0.1–0.5 Å/s, and the film thickness was monitored by a quartz crystal microbalance. The nominal thickness of the Ag film was set to 50–100 nm. During evaporation, the substrate temperature was maintained at room temperature to prevent damage to the ZnO nanorod structure and the PET substrate. After evaporation, the samples were cooled in a vacuum to room temperature before removal.

### 2.5. Sample Characterization

The UV-Vis absorption spectra were measured using an Ocean Optics HR4000 spectrophotometer (Orlando, FL, USA) in absorption mode. Measurements were performed at room temperature with a wavelength range of 300–800 nm, using air as the reference. The samples were positioned perpendicular to the incident beam path, and baseline correction was performed using blank substrates (ITO–glass or ITO-PET without ZnO nanorods) to account for substrate absorption contributions. X-ray diffraction (XRD) was used to analyze the crystal structure of the samples, with a scanning range of 2θ = 20–80°, a scanning rate of 2°/min, and a step size of 0.02°. Field emission scanning electron microscopy (FE-SEM, JEOL JSM-7800F, Tokyo, Japan) was employed to observe the surface morphology and microstructure of the samples. The operating voltage was set to 3–15 kV, and all samples were coated with a thin layer of gold for conductivity before observation to prevent charging effects. SERS measurements were conducted using a micro-Raman spectroscopy system (HORIBA Jobin Yvon, Paris, France). The micro-Raman system was equipped with confocal optics that focused the 633 nm He-Ne laser to the minimum achievable spot size through the 40× objective, ensuring a consistent illumination area for all SERS measurements and providing a spectral resolution of approximately 1 cm^−1^ and an integration time of 30 s. Aqueous solutions of R6G dissolved in ethanol at different concentrations (10^−3^ M to 10^−9^ M) were prepared as probe molecules. A quantity of 5 μL of R6G solution was dropped onto the SERS substrate surface, and measurements were performed after natural drying at room temperature. To verify the piezoelectric enhancement effect, bending tests were performed on samples on flexible ITO-PET substrates. The flexible substrate was carefully bent to a curvature radius of approximately 1–2 cm and fixed in the bent state using a clamp. SERS spectra of R6G at the same concentration were measured in both bent and unbent states, and the piezoelectric enhancement effect was evaluated by comparing the changes in signal intensity. Care was taken to avoid sample damage due to excessive deformation during bending. Software was used for spectral data analysis and graph plotting. The SERS enhancement factor (EF) was calculated using the formula: EF = (I_SERS_/C_SERS_)/(I_Raman_/C_Raman_), where I_SERS_ and I_Raman_ are the signal intensities under SERS conditions and normal Raman conditions, respectively, and C_SERS_ and C_Raman_ are the corresponding molecular concentrations.

## 3. Results and Discussion

### 3.1. Surface Morphology and Structural Characteristics of ZnO Nanorods Grown on Rigid Substrates

**Figure 1:**
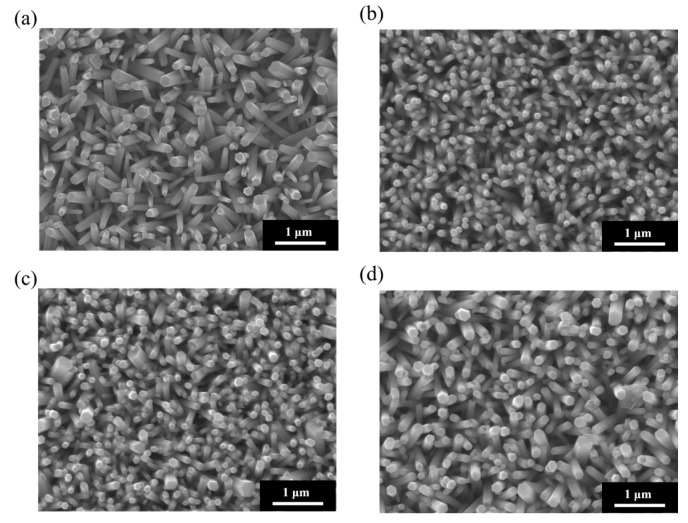
SEM surface morphologies of ZnO NRs grown on ITO–glass substrates at (**a**) 100 °C, (**b**) 150 °C, (**c**) 200 °C, and (**d**) 250 °C annealing temperatures. All samples were prepared using 4 seed layers.

Figure 1 shows the SEM surface morphologies of ZnO NRs grown on ITO–glass substrates using four seed layers and annealed at various temperatures (100 °C, 150 °C, 200 °C, and 250 °C). These SEM images clearly illustrate the significant impact of annealing temperature on the morphology, density, size, and alignment of the ZnO NRs. Samples grown at an annealing temperature of 100 °C (Figure 1a) exhibited relatively larger but lower-density hexagonal columnar structures. The NRs had diameters of approximately 200–300 nm, varying heights, and a somewhat scattered arrangement. However, their growth orientation was rather random, with many NRs appearing tilted or lying flat. This suggests that at lower annealing temperatures, the crystallinity and uniformity of the seed layer were insufficient, leading to inconsistent growth directions of the ZnO NRs [52,53]. As the annealing temperature increased to 150 °C (Figure 1b), the density of the ZnO NRs significantly increased, their diameters decreased to approximately 100–150 nm, and they showed a more uniform distribution and improved vertical alignment. Further increasing the annealing temperature to 200 °C (Figure 1c) led to continued improvement in the density and vertical alignment of the ZnO NRs. The nanorod diameters ranged from approximately 80 to 120 nm, and they were more densely packed and vertically oriented. When the annealing temperature reached 250 °C (Figure 1d), the ZnO NRs exhibited optimal morphological characteristics: uniform diameters (approximately 80–100 nm), consistent heights, the highest density, and nearly perfect vertical alignment for most NRs. The hexagonal structures at the tops of these NRs were more distinct, and their sidewalls were smooth, indicating significantly improved crystallinity. Furthermore, the inter-nanorod spacing was appropriate, approximately 30–50 nm, a structure beneficial for the uniform deposition of subsequent metal nanoparticles and providing sufficient space for analyte molecule adsorption. The influence of annealing temperature on the morphology of ZnO NRs can be attributed to several factors. Firstly, higher annealing temperatures promote the maturation and recrystallization of ZnO nanocrystals within the seed layer, reducing defects and grain boundaries, and thereby improving the crystallinity of the seed layer. Secondly, high-temperature annealing enhances the uniformity and orientation of the seed layer, providing more and more uniformly distributed nucleation sites, which favors the vertical growth of ZnO NRs. Finally, a high-quality seed layer can reduce secondary nucleation during subsequent hydrothermal growth, leading to a nanorod array with a more uniform size and orientation.

**Figure 2:**
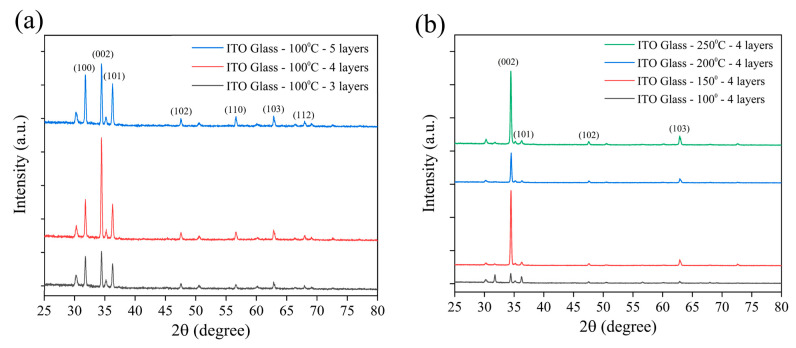
XRD analysis patterns of ZnO NRs on ITO–glass substrates: (**a**) the effect of different numbers of seed layers (3–5 layers) on the crystal structure of ZnO NRs at a fixed annealing temperature of 100 °C; (**b**) the effect of different annealing temperatures (100–250 °C) on the crystal structure of ZnO NRs under a fixed condition of 4 seed layers.

Figure 2 displays the XRD patterns of ZnO NRs grown on ITO–glass substrates, used to confirm their crystal structure and preferred orientation. All samples exhibited characteristic diffraction peaks consistent with the hexagonal wurtzite structure of ZnO [54]. The main diffraction peaks were observed at 2θ = 31.7°, 34.4°, 36.3°, 47.5°, 56.6°, and 62.9°, corresponding to the (100), (002), (101), (102), (110), and (103) planes of ZnO, JCPDS Card No. 36-1451, respectively [55,56]. Among these, the (002) peak showed a significantly higher intensity than the other peaks, indicating that the ZnO NRs possessed a preferred growth orientation along the c-axis direction. This aligns with the typical growth behavior of hexagonal wurtzite ZnO under hydrothermal growth conditions. Figure 2a compares the influence of varying seed layer numbers (three, four, and five layers) on the crystal structure of ZnO NRs while maintaining a fixed annealing temperature of 100 °C. It can be observed that as the number of seed layers increased, the intensity of the main diffraction peaks gradually enhanced, with a particularly significant increase in the relative intensity of the (002) peak. This suggests that increasing the number of seed layers facilitates the oriented growth of ZnO NRs along the c-axis, leading to a more ordered nanorod array [17]. When the seed layers reached five, the intensity ratio of the (002) peak to other diffraction peaks reached its maximum, indicating the highest c-axis orientation of the ZnO NRs at this condition. The increased number of seed layers likely provided more nucleation sites and a more uniform seed distribution, promoting the vertical growth of the ZnO NRs. Figure 2b investigates the effect of annealing temperature (100 °C, 150 °C, 200 °C, and 250 °C) on the crystal structure of ZnO NRs under a fixed condition of four seed layers. The results show that as the annealing temperature increased, the intensity of the (002) peak significantly strengthened. When the temperature reached 250 °C, the relative intensity of the (002) peak reached its maximum, while the relative intensities of other peaks, such as (100) and (101), significantly decreased. This indicates that higher annealing temperatures contribute to improving the crystallinity and orientation of the seed layer, thereby promoting the preferred c-axis-oriented growth of ZnO NRs [57,58]. Notably, in the sample annealed at 250 °C, the (002)/(101) peak intensity ratio reached its highest value, suggesting that ZnO NRs with optimal c-axis orientation were obtained under this condition. These highly oriented ZnO NRs are significant for subsequent modification with metal nanoparticles and SERS applications, as they can provide a uniform surface morphology and a good piezoelectric response, which are beneficial for SERS signal enhancement. This highly oriented ZnO nanorod structure is conducive to generating a consistent piezoelectric response, which is crucial for subsequent SERS signal enhancement via the piezoelectric effect. Morphological observations are highly consistent with the XRD analysis results, confirming the significant impact of annealing temperature on ZnO nanorod growth. Based on the SEM and XRD analyses, we conclude that ZnO NRs grown on ITO–glass substrates using four seed layers and annealed at 250 °C possess the best morphological and structural characteristics, including a high density, uniform size, good vertical orientation, and excellent crystallinity. This high-quality ZnO nanorod array lays a solid foundation for subsequent metal functionalization and SERS applications. These morphological results are also consistent with trends reported in the literature, where higher annealing temperatures generally promote the vertical oriented growth and improve the crystallinity of ZnO NRs. Importantly, an annealing temperature of 250 °C remains within the thermal tolerance range of PET substrates (approximately 260–265 °C), which provides feasible technical parameters for preparing similar ZnO nanorod structures on flexible PET substrates, facilitating the subsequent development of piezoelectric-enhanced SERS sensors on flexible substrates.

### 3.2. Structural and Surface Morphology Characteristics of ZnO Nanorods on Flexible Substrates

**Figure 3:**
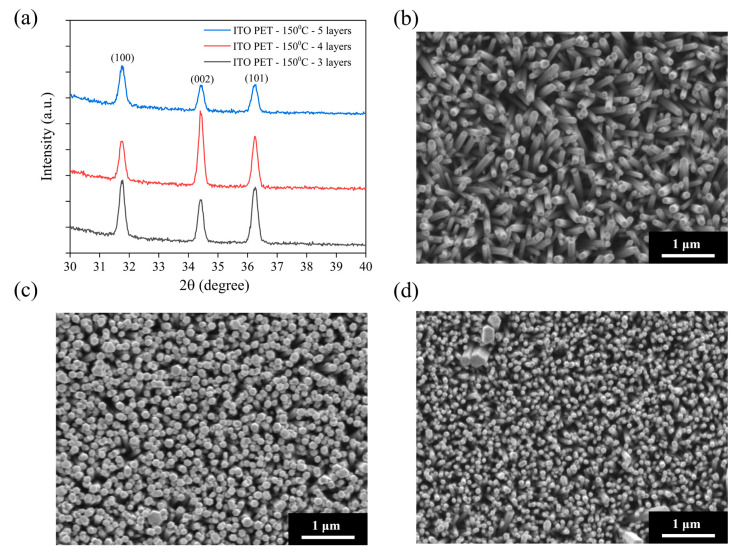
Structural and morphological analysis of ZnO NRs on flexible ITO-PET substrates: (**a**) XRD patterns of ZnO NRs prepared with different numbers of seed layers (3–5 layers) at an annealing temperature of 150 °C; SEM surface morphology images of ZnO NRs grown on ITO-PET substrates with (**b**) 3, (**c**) 4, and (**d**) 5 seed layers. All samples were annealed at 150 °C.

To compare the influence of rigid (ITO–glass) and flexible (ITO-PET) substrates on ZnO nanorod growth, we fabricated ZnO nanorod arrays on ITO-PET substrates under similar conditions. Considering the thermal resistance limitations of the PET substrate, the annealing temperature was set at 150 °C, well below the glass transition temperature of PET, to ensure the structural integrity of the substrate during the process. Figure 3a shows the XRD patterns of ZnO NRs grown on ITO-PET substrates using different numbers of seed layers (three, four, and five layers) and annealed at 150 °C. Compared to ZnO NRs on ITO–glass substrates, those grown on PET substrates exhibited distinctly different crystal structural characteristics. All samples displayed the main diffraction peaks of hexagonal wurtzite ZnO, including (100), (002), and (101), but with significantly different relative intensity ratios. On ITO-PET substrates, the intensities of the (100) and (101) peaks were significantly higher than that of the (002) peak. This contrasts sharply with the ITO–glass substrates, where the (002) peak was dominant. This indicates that ZnO NRs grown on flexible PET substrates exhibit less c-axis-preferential orientation and show more non-vertical growth patterns. This structural difference may stem from the distinct surface properties of PET substrates compared to glass, as well as potential minor deformations in the flexible substrate during processing. As the number of seed layers increased from three to five, the relative intensity of the (002) peak slightly increased, suggesting that increasing the number of seed layers contributes to improving the c-axis orientation of the ZnO NRs. However, even with five seed layers, the (002) peak intensity remained notably lower than that of the (100) and (101) peaks, illustrating the difficulty in achieving highly oriented ZnO nanorod structures on flexible substrates like those on glass substrates.

Figure 3b–d shows the SEM morphological images of ZnO NRs grown on ITO-PET substrates using different numbers of seed layers (three, four, and five layers) and annealed at 150 °C. These images are consistent with the XRD results, showing distinct morphological features compared to NRs on ITO–glass substrates. The samples prepared with three seed layers (Figure 3b) exhibited short and tilted nanorod structures, with diameters of approximately 80–120 nm, shorter lengths (around 300–400 nm), and a moderate distribution density. The orientation of these NRs showed clear randomness, with most forming an angle with the substrate and very few being perfectly perpendicular. This aligns with the XRD analysis, where the (100) and (101) peaks were stronger than the (002) peak. As the seed layers increased to four (Figure 3c), the nanorod density significantly increased, their diameters decreased to approximately 60–100 nm, and they became more densely packed. Although the orientation still showed some randomness, more NRs perpendicular to the substrate could be observed, indicating that increasing the number of seed layers helped improve the orientation of the ZnO NRs. Additionally, the top morphology of these NRs was more uniform, mostly displaying hexagonal features, suggesting an improvement in crystallinity. When the seed layers reached five (Figure 3d), the nanorod density further increased, and their diameters further decreased to approximately 50–80 nm, forming a high-density nanorod array. While these NRs still showed some tilting, their overall vertical orientation was improved, consistent with the trend of increased relative (002) peak intensity in XRD.

Comparison of ZnO NRs grown on ITO–glass and ITO-PET substrates revealed significant substrate-dependent variations in structure and morphology. ZnO NRs on ITO–glass substrates exhibited a strong c-axis-preferred orientation with long, upright columnar structures perpendicular to the substrate surface, while those on ITO-PET substrates primarily displayed (100) and (101) orientations with shorter, more dispersed arrangements, often appearing tilted. Under similar preparation conditions, NRs on ITO-PET substrates demonstrated smaller diameters but a higher density compared to those on ITO–glass substrates, forming more compact nanorod arrays. These structural differences reflect the decisive influence of substrate surface properties and structural stability on ZnO nanorod growth direction and morphology. These differences may arise from several factors. First, the surface energy and lattice properties of the PET substrate differ from those of glass, affecting the adhesion and crystallization of the seed layer. Second, the PET substrate may undergo minor thermal expansion and deformation during heat treatment, influencing the uniformity of the seed layer and subsequent nanorod growth. Finally, the flexible nature of the PET substrate might lead to slight bending or deformation during processing, resulting in an uneven distribution of growth stress, thereby affecting the growth orientation of the NRs. The observed structural differences on PET substrates can be attributed to several factors related to the flexible substrate’s thermal response. During the 150 °C annealing process, PET substrates experienced slight thermal deformation due to their inherent flexibility and thermal expansion properties, even though the temperature was well below PET’s glass transition point (~260–265 °C). This thermal-induced warping created locally varying surface orientations that influenced ZnO seed layer crystallization and subsequent nanorod growth directions. The thermal expansion mismatch between the ITO coating and PET substrate may also have introduced interfacial stresses affecting crystal nucleation patterns, resulting in the more diverse nanorod orientations observed compared to the uniform vertical growth achieved on rigid glass substrates.

### 3.3. Morphological Characteristics of PLIP-Fabricated Au-ZnO Nanocomposites

**Figure 4:**
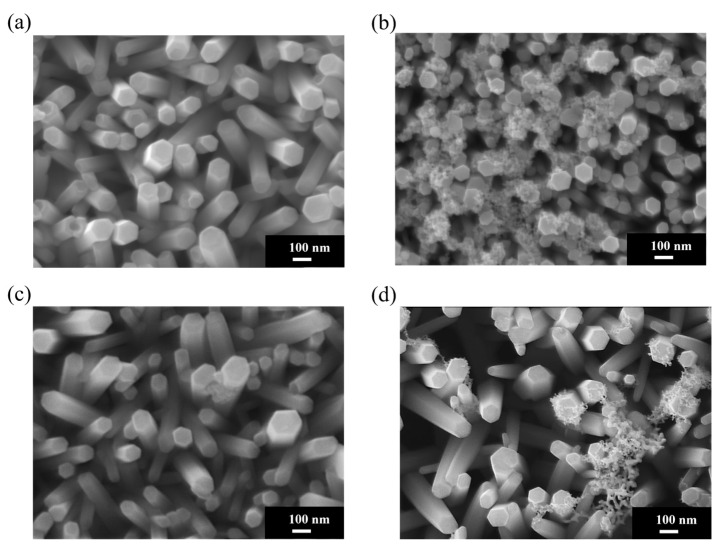
SEM morphological analysis of ZnO NRs on different substrates before and after Au nanoparticle deposition using PLIP method: (**a**) pristine ZnO NRs and (**b**) Au-ZnO NCPs on an ITO–glass substrate; (**c**) pristine ZnO NRs and (**d**) Au-ZnO NCPs on an ITO-PET substrate.

Figure 4a shows an SEM image of pristine ZnO NRs on an ITO–glass substrate. It clearly shows that the ZnO NRs exhibit a regular hexagonal columnar structure with smooth surfaces and diameters of approximately 80–100 nm and that they are neatly arranged, with most being perpendicular to the substrate. These NRs present distinct hexagonal top facets, reflecting the hexagonal wurtzite crystal structure of ZnO. Figure 4b shows the Au-ZnO NCP structure on the same ITO–glass substrate after Au nanoparticle deposition by the PLIP method. The SEM image clearly shows that many Au nanoparticles are uniformly attached to the surface of the ZnO NRs. These nanoparticles are spherical or near-spherical, with an average diameter of approximately 10–20 nm; uniformly distributed; and with a moderate density. Notably, the Au nanoparticles are deposited not only on the top but also on the sidewalls of the ZnO NRs, forming an omnidirectional coating. Additionally, a certain amount of Au nanoparticles is deposited in the voids between the ZnO NRs, which may contribute to the formation of more “hot spot” regions, positively impacting SERS signal enhancement. Figure 4c shows an SEM image of pristine ZnO NRs on an ITO-PET substrate. Compared to the NRs on ITO–glass substrates, the ZnO NRs on ITO-PET have smaller diameters (approximately 60–80 nm), are relatively shorter in length, and exhibit dispersed orientations, with many NRs appearing tilted. Simultaneously, the hexagonal morphological features of these NRs are less distinct, suggesting that their crystallinity might be slightly lower than that of NRs on glass substrates. Figure 4d shows the Au-ZnO NCP structure on an ITO-PET substrate after Au nanoparticle deposition by the PLIP method. Au nanoparticles were successfully deposited on the ZnO nanorod surfaces, but their distribution characteristics differed compared to the ITO–glass substrate. On the PET substrate, the Au nanoparticle size distribution was broader (approximately 10–30 nm), and in some areas, larger Au aggregates or clustered structures (approximately 50–100 nm) appeared. These clustered structures were primarily distributed in the gaps between the NRs rather than uniformly attached to their surfaces. Furthermore, in certain regions on the PET substrate, the Au nanoparticles exhibited networked or chained aggregated structures. This might be related to the unique arrangement of ZnO NRs on the PET substrate. While this aggregated structure reduces the uniformity of Au nanoparticle distribution, it may form more nanogaps and hot spot regions, potentially further enhancing the SERS effect.

### 3.4. Morphological Characteristics of Thermal-Evaporation-Fabricated Ag-ZnO Nanocomposites

**Figure 5:**
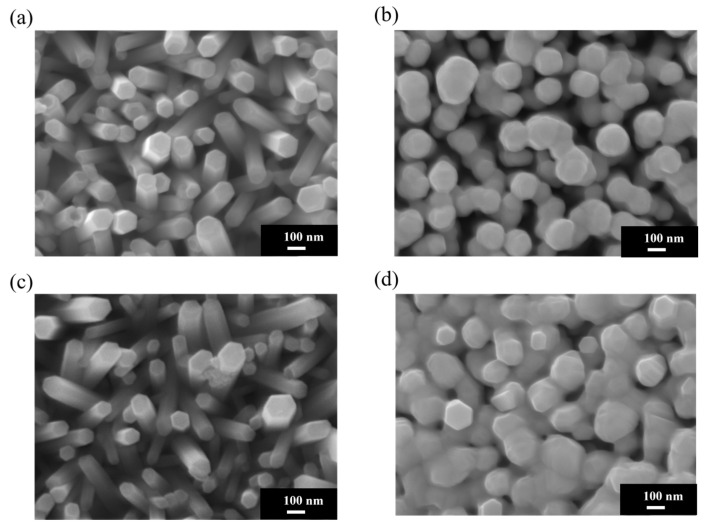
SEM morphological analysis of ZnO NRs on different substrates before and after Ag deposition using thermal evaporation: (**a**) pristine ZnO NRs and (**b**) Ag-ZnO NCPs on an ITO–glass substrate; (**c**) pristine ZnO NRs and (**d**) Ag-ZnO NCPs on an ITO-PET substrate.

Figure 5a displays an SEM image of pristine ZnO NRs on an ITO–glass substrate, revealing regularly arranged hexagonal columnar ZnO NRs with diameters of approximately 80–100 nm, mostly perpendicular to the substrate surface. These NRs possess smooth surfaces and distinct hexagonal tops, reflecting their excellent crystalline quality. Figure 5b showcases the Ag-ZnO NCPs on ITO–glass substrates after Ag deposition by thermal evaporation. Notably, thermal evaporation is inherently a technique for depositing continuous metal thin films, rather than forming dispersed nanoparticles. The SEM image clearly shows that silver primarily covers the tops and upward-facing surfaces of the ZnO NRs in the form of a thin film, making the originally distinct hexagonal columnar structures appear more rounded. This coverage pattern is completely different from the dispersed Au nanoparticles produced by the laser method; the Ag film exhibits a more continuous coverage, forming a silver coating of an approximately 20–30 nm thickness on the nanorod tops. Figure 5c shows an SEM image of pristine ZnO NRs on an ITO-PET substrate. As previously mentioned, ZnO NRs on PET substrates exhibit smaller diameters (approximately 60–80 nm), a higher density, and more dispersed orientations, with many NRs appearing tilted to varying degrees. This structural characteristic provides a different foundation for subsequent Ag thin film deposition compared to glass substrates. Figure 5d shows the Ag-ZnO NCPs formed after Ag deposition on an ITO-PET substrate by thermal evaporation. As with the glass substrates, silver primarily covers the tops and upward-exposed surfaces of the ZnO NRs as a thin film. However, due to the more dispersed and tilted orientation of NRs on the PET substrate, the distribution of the silver film also exhibited a more complex coverage pattern. Many tilted nanorod sidewalls also received silver atoms, leading to partial sidewall coverage and giving the entire composite structure a more intricate three-dimensional network characteristic. On the PET substrate, the silver film formed distinctive bridging structures connecting adjacent NRs due to their tilted arrangement, creating nanoscale gaps and crevices that serve as intense electromagnetic field enhancement regions. This interconnected Ag film architecture transforms the originally dispersed ZnO nanorod array into a cohesive network, facilitating both electron transport and electromagnetic field propagation throughout the composite structure. The resulting three-dimensional metallic network provides multiple coupling pathways that may enhance SERS performance beyond that achievable with isolated nanorod–metal interfaces.

### 3.5. Optical Properties of Metal–ZnO Nanocomposites

**Figure 6:**
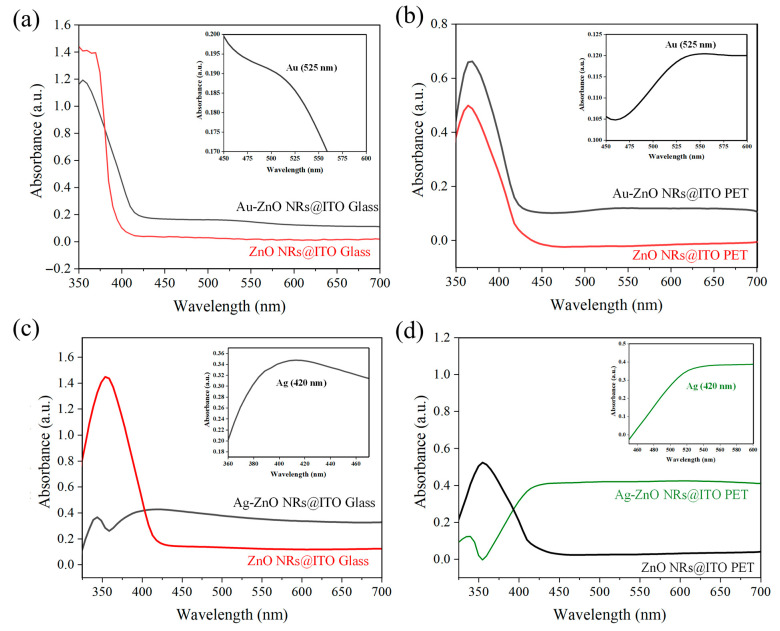
UV-Vis absorption spectra of ZnO NRs and metal–ZnO structures on different substrates. (**a**) ZnO NRs (red line) and Au-ZnO NCPs (black line) on ITO–glass substrate; (**b**) ZnO NRs (red line) and Au-ZnO NCPs (black line) on ITO-PET substrate; (**c**) ZnO NRs (red line) and Ag-ZnO NCPs (black line) on ITO–glass substrate; (**d**) ZnO NRs (black line) and Ag-ZnO NCPs (green line) on ITO-PET substrate. The insets in each figure show magnified views of the characteristic LSPR absorption peaks of the metal nanostructures.

Ultraviolet–visible (UV-Vis) absorption spectroscopy is a crucial tool for characterizing the optical properties of metal–semiconductor composite structures, providing essential information about material band structure, LSPR characteristics, and interfacial interactions. Figure 6 shows the absorption spectra of ZnO NRs on different substrates and their composites with Au or Ag, reflecting the influence of substrate type, metal species, and deposition method on the optical properties of the composite structures. From the red curves in all samples in Figure 6, it can be observed that pristine ZnO NRs (unmodified with metal) exhibit strong absorption in the UV region (approximately 370–380 nm), corresponding to the band-to-band transition of ZnO (band gap approximately 3.37 eV). Both ZnO NRs on glass and PET substrates showed similar UV absorption characteristics, but with slight differences in intensity and precise peak positions. These variations may be related to the differences in the size, density, and crystalline quality of the ZnO NRs on the two substrates. ZnO NRs on ITO–glass substrates (red lines in Figure 6a,c) show a relatively broad absorption band around 370 nm, while ZnO NRs on ITO-PET substrates (red line in Figure 6b and black line in Figure 6d) show a sharper absorption peak at 375–380 nm. This difference might reflect the distinct crystallographic orientation and defect states of ZnO NRs on PET substrates. Additionally, the absorption intensity of ZnO NRs on ITO-PET substrates was relatively lower, which could be attributed to their smaller diameter and shorter length. In the visible light region (400–700 nm), all pristine ZnO nanorod samples exhibit low absorption, consistent with the intrinsic nature of ZnO as a wide-bandgap semiconductor. From an optical transparency perspective, these ZnO nanorod structures possess good transparency in the visible light region, which is beneficial for their application as transparent SERS substrates. After depositing Au nanoparticles using PLIP, the optical properties of the ZnO NRs underwent significant changes. As shown in Figure 6a,b, the Au-ZnO composite structures (black lines) exhibited enhanced absorption across the entire visible light region. Notably, a characteristic LSPR absorption peak appeared around 525 nm, which is clearly amplified in the insets of each figure.

The inset in Figure 6a shows that the Au-ZnO composite structure on the ITO–glass substrate presented typical Au nanoparticle SPR absorption characteristics in the 450–600 nm wavelength range. The absorbance gradually increased from approximately 0.1735 at 450 nm, reaching a more distinct peak (approximately 0.1825) near 525 nm, then slightly decreasing at longer wavelengths. This relatively gentle SPR feature may be related to the uniform distribution of Au nanoparticles on the glass substrate. In contrast, the inset in Figure 6b indicates that the Au-ZnO composite structure on the ITO-PET substrate exhibited a more pronounced SPR peak, with absorbance significantly increasing from approximately 0.105 at 450 nm to about 0.120 at 525 nm, an increase of approximately 14%, and maintaining a higher absorption plateau at longer wavelengths. This stronger LSPR characteristic can be attributed to the higher distribution density and more diverse nanostructures (including nanoparticle aggregates and networked structures) of Au nanoparticles on the PET substrate. Furthermore, changes were observed in the UV absorption band of the Au-ZnO NCPs, including an enhancement in intensity and a slight redshift. This suggests that the introduction of Au nanoparticles not only increased absorption in the visible light region but also potentially influenced the band structure and exciton absorption characteristics of ZnO through interfacial interactions.

Ag-ZnO composite structures prepared by thermal evaporation exhibited distinctly different optical properties compared to Au-ZnO, as shown in Figure 6c,d. The inset in Figure 6c clearly shows that the Ag-ZnO composite structure on the ITO–glass substrate presented a characteristic SPR absorption peak at approximately 420 nm. The absorbance rose from approximately 0.345 at 385 nm to about 0.353 at 420 nm, then slightly decreased at longer wavelengths. This SPR characteristic was relatively weak and partially overlapped with the ZnO absorption band, likely because thermal evaporation forms a continuous Ag thin film coating rather than dispersed nanoparticles. In contrast, the Ag-ZnO composite structure on the ITO-PET substrate (represented by the green line in Figure 6d) showed a stronger and more pronounced LSPR absorption, with its inset revealing a significant increase in absorbance from approximately 0.0 at 350 nm to about 0.35 at 420 nm, an extremely notable increase. This strong LSPR characteristic might originate from the unique tilted arrangement of ZnO NRs on the PET substrate, which allowed the Ag film to form a more complex three-dimensional network structure, significantly enhancing its plasmon resonance effect. Notably, in the long wavelength region of visible light (500–700 nm), the Ag-ZnO composite structure on the ITO-PET substrate maintained a high absorption value (approximately 0.4). This broadband absorption characteristic may be beneficial for enhancing Raman scattering, especially when using 532 nm or 633 nm excitation sources.

### 3.6. Crystal Characterization of Metal–ZnO Composite Structures

**Figure 7:**
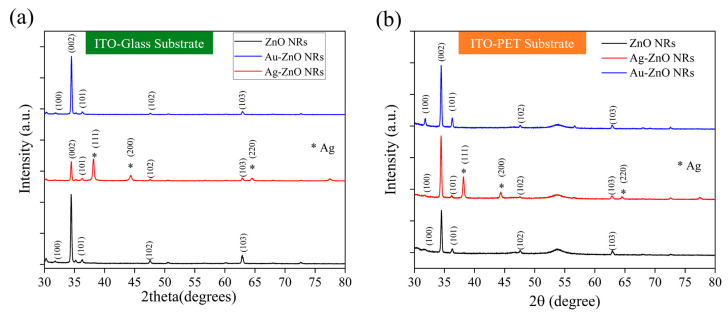
XRD analysis of ZnO nanorod crystal structures before and after metal modification on different substrates. (**a**) ZnO NRs (black line), Au-ZnO NCPs (red line), and Ag-ZnO NCPs (blue line) on an ITO–glass substrate, with all samples annealed at 250 °C; (**b**) ZnO nanorods (black line), Au-ZnO NCPs (blue line), and Ag-ZnO composite structures (red line) on an ITO-PET substrate, with all samples annealed at 150 °C.

Figure 7a shows the XRD patterns of ZnO NRs grown on an ITO–glass substrate and the NCPs after metal modification. The pristine ZnO NRs (black line) exhibited characteristic diffraction peaks consistent with the hexagonal wurtzite structure, primarily including peaks at 2θ = 31.7°, 34.4°, 36.3°, 47.5°, 56.6°, and 62.9°, corresponding to the (100), (002), (101), (102), (110), and (103) planes of ZnO, respectively. Among these, the (002) peak intensity was significantly higher than the other peaks, indicating a preferential c-axis-oriented growth of ZnO NRs on the ITO–glass substrate, which is consistent with the vertically aligned nanorod morphology observed in previous SEM analyses. After deposition of Au nanoparticles via laser-induced photolysis (red line), the main diffraction peaks of ZnO remained unchanged, indicating that the deposition of Au nanoparticles did not disrupt the fundamental crystal structure of the ZnO NRs. Notably, the relative intensity of the (002) peak further increased, which might suggest that the presence of Au nanoparticles helps enhance the c-axis orientation of ZnO or improve the diffraction capability of specific crystal planes. However, no distinct Au characteristic diffraction peaks were observed in this XRD pattern. This could be attributed to the small size of the Au nanoparticles (10–20 nm), the low loading amount, or good dispersion, making their diffraction signal below the detection limit.

In contrast, samples with Ag deposited by thermal evaporation (blue line) not only retained the main diffraction peaks of ZnO but also showed several distinct Ag characteristic diffraction peaks at 2θ = 38.1°, 44.3°, 64.4°, and 77.5°, corresponding to the (111), (200), (220), and (311) planes of face-centered cubic Ag (these peaks are marked with an asterisk (*) in the figure). These clear Ag diffraction peaks confirm the successful deposition of the Ag film on the ZnO nanorod surfaces and indicate that the Ag film formed by thermal evaporation possesses good crystallinity. Furthermore, the high intensity of the Ag (111) peak suggests that the deposited Ag film may have a preferential (111) orientation, consistent with the typical growth characteristics of face-centered cubic metal materials. Another significant finding is that after Ag modification, the intensity of the ZnO (002) peak relatively weakened, while the (100) and (101) peaks relatively strengthened. This change suggests that the deposition of the Ag film might have partially covered the top (002) planes of the ZnO NRs, reducing their diffraction signal, while increasing the relative contribution from the sidewall planes. This aligns with the SEM observation that Ag preferentially covers the nanorod tops.

Figure 7b displays the XRD patterns of ZnO NRs and their metal-modified composite structures on an ITO-PET substrate. Compared to the ITO–glass substrate, the pristine ZnO NRs (black line) on the PET substrate exhibited distinctly different crystal orientation characteristics. Although the main diffraction peaks of ZnO were still observed, the relative intensities of the (100) and (101) peaks were significantly higher than the (002) peak, indicating that ZnO NRs on PET substrates had more non-c-axis-oriented growth, which is consistent with the tilted nanorod arrangement observed in previous SEM analyses. The Au-modified samples (blue line) showed that the main diffraction peak pattern of ZnO remained unchanged, but the overall intensity was enhanced, suggesting that the deposition of Au nanoparticles might have improved the crystallinity or diffraction efficiency of ZnO. Similar to the glass substrate case, characteristic diffraction peaks of Au nanoparticles were not prominent on the PET substrate, likely for the same reasons mentioned above. For the Ag-modified samples (red line), clear Ag characteristic diffraction peaks were also observed at 2θ = 38.1°, 44.3°, and 64.4°, corresponding to the (111), (200), and (220) planes of Ag, respectively. However, compared to the glass substrate, the Ag diffraction peak intensities on the PET substrate were relatively weaker, and the (200) peak was more pronounced, indicating that the substrate type might influence the crystal orientation and quality of the Ag film. Of particular note is that on the PET substrate, the intensity of the ZnO (002) peak was significantly enhanced after Ag modification, which is contrary to the trend observed on the glass substrate. This phenomenon may be attributed to the unique tilted arrangement of ZnO NRs on the PET substrate, which resulted in less coverage of the c-axis planes during Ag film deposition. Instead, it might have enhanced the diffraction capability of the c-axis orientation through stress effects or interfacial interactions.

### 3.7. SERS Performance of R6G Molecules on Rigid Substrates

**Figure 8:**
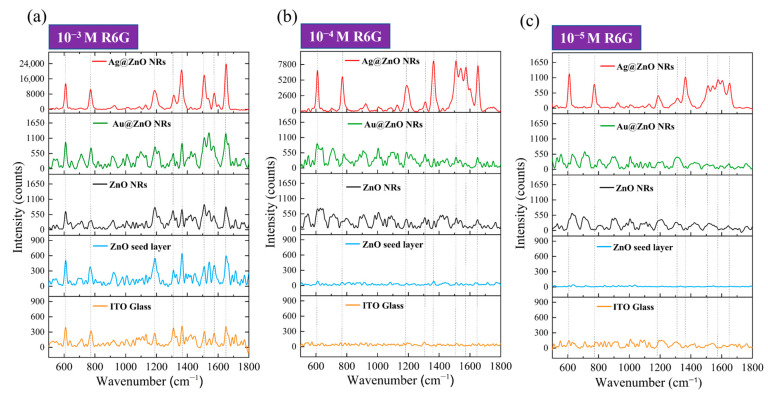
Raman spectral responses of R6G molecules on different SERS substrates on ITO–glass at (**a**) 10^−3^ M R6G, (**b**) 10^−4^ M R6G, and (**c**) 10^−5^ M R6G.

In this study, we used R6G as a probe molecule to systematically compare the SERS enhancement effects of different substrates on R6G molecules. Figure 8 presents the Raman spectral responses of R6G molecules at three different concentrations, ranging from 10^−3^ M to 10^−5^ M, on ITO–glass substrates. First, by analyzing the Raman spectra of R6G on a pristine ITO–glass substrate (bottom orange line in each sub-figure of Figure 8), we can identify the main characteristic peaks of the R6G molecule. At a high concentration of 10^−3^ M (Figure 8a, orange line), the primary characteristic peaks of R6G are located at 612 cm^−1^ (C-C-C ring in-plane bending vibration), 774 cm^−1^ (C-H out-of-plane bending vibration), 1186 cm^−1^ (C-H in-plane bending vibration), 1311 cm^−1^ (C-O-C stretching vibration), 1364 cm^−1^ (aromatic ring C-C stretching vibration), 1510 cm^−1^ (aromatic ring C-C stretching vibration), and 1652 cm^−1^ (C=C stretching vibration) [17]. These peak positions are consistent with literature reports for R6G characteristic peaks and are indicated by dashed lines in the figure. However, when the concentration decreased to 10^−4^ M and 10^−5^ M (Figure 8b,c, orange lines), clear R6G characteristic peaks were almost indistinguishable on the pristine ITO–glass substrate; only faint background signals and noise were observed.

The blue lines in Figure 8 represent the SERS response of the ZnO seed layer to R6G. At the 10^−3^ M concentration (Figure 8a, blue line), the ZnO seed layer showed a weak SERS enhancement effect, allowing for the identification of some R6G characteristic peaks, but with limited signal intensity. When the concentration dropped to 10^−4^ M (Figure 8b, blue line), the Raman signal significantly weakened, with only a few stronger characteristic peaks being discernible. At the 10^−5^ M concentration (Figure 8c, blue line), almost no clear R6G characteristic peaks were observed. This indicates that while the ZnO seed layer provided some surface enhancement, its enhancement factor was low, limiting its detection. The black lines represent the SERS response of pristine ZnO NRs to R6G. Compared to the ZnO seed layer, ZnO NRs exhibited a stronger enhancement effect at all concentrations. At the 10^−3^ M concentration (Figure 8a, black line), all R6G characteristic peaks were clearly visible, and the signal intensity was significantly higher than that of the ZnO seed layer and pristine substrate. Even at low concentrations of 10^−4^ M and 10^−5^ M (Figure 8b,c, black lines), the main characteristic peaks, particularly at 612 cm^−1^, 774 cm^−1^, and 1652 cm^−1^, could still be identified. This suggests that the ZnO nanorod structure provided a significant increase in surface area and a potential chemical enhancement effect, effectively improving SERS performance. The green lines illustrate the SERS response of the Au-ZnO composite structure to R6G. The Au nanoparticles deposited via laser-induced photolysis further enhanced the SERS performance of the ZnO NRs. At the 10^−3^ M concentration (Figure 8a, green line), all R6G characteristic peaks were not only clearly visible but also had significantly higher peak intensities than those of pristine ZnO NRs. Notably, the aromatic ring stretching vibration peaks at 1364 cm^−1^ and 1510 cm^−1^ showed the most significant intensity increase. At the 10^−4^ M concentration (Figure 8b, green line), Au-ZnO still clearly presented all characteristic peaks, with sharper peak shapes and relatively lower background noise. Even at the low concentration of 10^−5^ M (Figure 8c, green line), the main characteristic peaks were still discernible, and the signal intensity was clearly superior to that of pristine ZnO NRs, indicating that Au nanoparticles effectively enhanced SERS detection sensitivity. The enhancement effect of the Au-ZnO composite structure primarily originates from two aspects: on the one hand, the LSPR effect generated by Au nanoparticles significantly enhances the localized electromagnetic field, thereby intensifying the Raman scattering signal; on the other hand, the interface formed between Au and ZnO may promote charge transfer processes, enhancing the chemical enhancement mechanism. Additionally, the omnidirectional coverage of Au nanoparticles (as shown in SEM) creates abundant “hot spot” regions, further improving SERS detection sensitivity.

The red lines show the SERS response of the Ag-ZnO composite structure to R6G, which performed the best among all substrates. At the 10^−3^ M concentration (Figure 8a, red line), the SERS signal intensity produced by Ag-ZnO was significantly higher than for all other substrates, with the main characteristic peak intensities 2–3 times those of Au-ZnO and over 10 times those of pristine ZnO NRs. Particularly, the peaks at 612 cm^−1^, 1364 cm^−1^, and 1652cm^−1^ were extremely prominent. At the 10^−4^ M concentration (Figure 8b, red line), even with a 10-fold reduction in concentration, Ag-ZnO still provided an extremely strong SERS enhancement effect, with all characteristic peaks clearly discernible and relatively low background noise. Even at the low concentration of 10^−5^ M (Figure 8c, red line), the main characteristic peaks were still clearly identifiable, especially those at 612 cm^−1^ and 774 cm^−1^. The excellent SERS performance exhibited by the Ag-ZnO composite structure can be attributed to several key factors. Firstly, Ag possesses a stronger LSPR effect in the visible light region (especially near the 532 nm excitation wavelength) than Au, generating a stronger electromagnetic field enhancement; secondly, the Ag thin film coating formed by thermal evaporation created a continuous metal–semiconductor interface on the ZnO nanorod surface, which might provide more efficient interfacial charge transfer; thirdly, the Ag thin film bridging structures observed in SEM might create additional nanogap “hot spots,” further enhancing the SERS signal. Furthermore, XRD analysis showed that the Ag film has good crystallinity, which may contribute to generating more stable and stronger plasmon resonance effects. Based on the SERS data in Figure 8, we can estimate the SERS EFs for different substrates. Taking the characteristic peak at 1364 cm^−1^ for calculation, with the R6G signal at 10^−3^ M on pristine ITO–glass as a reference, rough estimations yielded an EF of approximately 10^2^−10^3^ for ZnO NRs, approximately 10^4^−10^5^ for Au-ZnO composite structures, and an EF of 10^5^−10^6^ for Ag-ZnO composite structures. The EF calculations were performed using the standard formula: EF = (I_SERS_/C_SERS_)/(I_Raman_/C_Raman_), where I_SERS_ and I_Raman_ represent the Raman signal intensities of the 1364 cm^−1^ peak from SERS substrates and pristine ITO–glass respectively, while C_SERS_ and C_Raman_ are the corresponding R6G concentrations. For example, Au-ZnO substrates showing detectable signals at 10^−5^ M R6G were compared against the reference signal from pristine ITO–glass at 10^−3^ M R6G. The concentration ratio (C_Raman_/C_SERS_ = 10^−3^/10^−5^ = 100) combined with the observed signal intensity enhancement yielded the reported EF range of 10^4^–10^5^ for Au-ZnO composite structures

### 3.8. SERS Performance of R6G Molecules on Flexible Substrates

**Figure 9:**
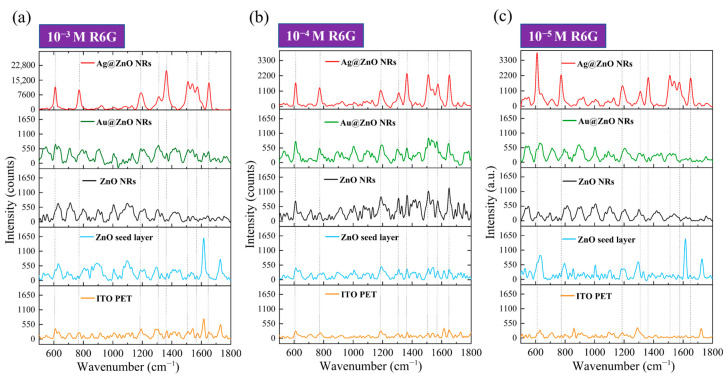
Raman spectral responses of R6G molecules on different SERS substrates on flexible ITO-PET at (**a**) 10^−3^ M R6G, (**b**) 10^−4^ M R6G, and (**c**) 10^−5^ M R6G.

Figure 9 shows the Raman spectral responses of R6G molecules at three concentrations (10^−3^ M to 10^−5^ M) on different SERS substrates on ITO-PET. First, by comparing the Raman spectra of R6G on a pristine ITO-PET substrate (bottom orange line in each sub-figure of Figure 9), we observed characteristic R6G peaks similar to those on the ITO–glass substrate, located at 612 cm^−1^, 774 cm^−1^, 1186 cm^−1^, 1311 cm^−1^, 1364 cm^−1^, 1510 cm^−1^, and 1652 cm^−1^. However, at the 10^−3^ M concentration (Figure 9a, orange line), the intensity of the R6G characteristic peaks on the ITO-PET substrate was significantly higher than on the ITO–glass substrate; in particular, the C=C stretching vibration peak at 1652 cm^−1^ showed a stronger signal. This difference might originate from the distinct surface properties of the PET substrate, or slight bending of the flexible substrate during the measurement could have produced some surface enhancement. At lower concentrations (Figure 9b,c, orange lines), the R6G signal on the pristine ITO-PET substrate rapidly diminished. Weak characteristic peaks could still be observed at 10^−4^ M, whereas the signal at this concentration on the ITO–glass substrate had almost completely disappeared. This further suggests that flexible PET substrates may possess inherent properties favorable for SERS enhancement.

Unlike the case on ITO–glass substrates, the ZnO seed layer on ITO-PET substrates exhibited significantly enhanced SERS performance. As shown by the blue curves in Figure 9, the ZnO seed layer provided considerable SERS enhancement at all three concentrations. It is particularly noteworthy that at the 10^−3^ M and 10^−5^ M concentrations (Figure 9a,c, blue lines), the peak intensity at 1652 cm^−1^ was unusually high, even surpassing some other substrates. This peculiar phenomenon might be related to the crystal orientation and surface morphology of the ZnO seed layer on the PET substrate, or it could reflect unique interactions between the seed layer and the flexible substrate. The ZnO seed layer at a low concentration of 10^−5^ M (Figure 9c, blue line) could still produce clear SERS signals, especially around 1652 cm^−1^, demonstrating performance characteristics entirely different from those on rigid substrates. This difference might imply that the ZnO seed layer on flexible substrates possesses unique surface or interfacial properties that favor R6G molecule adsorption and Raman signal enhancement. Pristine ZnO NRs on ITO-PET substrates (black lines in Figure 9) also showed a different SERS performance compared to those on ITO–glass. While the overall signal intensity was slightly lower than that of the corresponding structures on rigid substrates, the signal quality and peak shape characteristics were improved. At the 10^−3^ M concentration (Figure 9a, black line), all R6G characteristic peaks were clearly visible, with sharper peak shapes and relatively lower background noise. At the 10^−4^ M and 10^−5^ M concentrations (Figure 9b,c, black lines), although the signal intensity decreased, the main characteristic peaks were still identifiable, particularly those at 612 cm^−1^, 774 cm^−1^, and1364 cm^−1^. Unlike ITO–glass substrates, these peaks on flexible substrates showed better signal retention, with less signal weakening when the concentration was reduced by 100 times. This indicates that ZnO NRs on flexible substrates might have better detection sensitivity for low-concentration molecules. This difference in performance might stem from the structural variations we previously observed in SEM and XRD analyses: ZnO NRs on ITO-PET substrates exhibit more mixed orientations and smaller sizes. This unique structure may provide a wider variety of molecular adsorption sites and enhancement mechanisms in Raman detection.

The Au-ZnO NCPs deposited by the PLIP method on ITO-PET substrates (green lines in Figure 9) exhibited a clear SERS enhancement effect, but their relative advantage was somewhat reduced compared to the corresponding structures on ITO–glass. At the 10^−3^ M concentration (Figure 9a, green line), the SERS signal intensity produced by Au-ZnO on ITO-PET was lower than that of the corresponding structure on ITO–glass, but the peak shapes were clearer and the background was lower. It should be noted that spectra obtained on PET substrates occasionally show additional peaks around 1730 cm^−1^, which correspond to C=O stretching vibrations from the underlying PET substrate rather than R6G signals. These substrate-related contributions become apparent in samples with weaker SERS enhancement where the substrate signal is not masked by enhanced analyte peaks. Our quantitative analysis focused exclusively on the characteristic R6G peaks at 612, 774, 1186, 1311, 1364, 1510, and 1652 cm^−1^. At lower concentrations (Figure 9b,c, green lines), the Au-ZnO NCP was still able to effectively enhance the Raman signal of R6G, especially for the characteristic peaks at 612 cm^−1^, 774 cm^−1^, and 1364cm^−1^. Notably, the signal retention at the 10^−5^ M concentration was relatively good, indicating that Au nanoparticles also provided an effective SERS enhancement mechanism on flexible substrates. This performance difference might be related to the unique distribution of Au nanoparticles on PET substrates, as observed in our SEM analysis: Au nanoparticles on PET substrates tended to form more clusters and networked structures, rather than uniformly dispersed single nanoparticles. This structural difference may affect the plasmon resonance effect and “hot spot” distribution, thereby influencing SERS performance.

Among all SERS substrates on flexible substrates, the Ag-ZnO NCPs (red lines in Figure 9) exhibited the most excellent performance, similar to the case on rigid substrates. However, the Ag-ZnO NCPs on ITO-PET substrates not only outperformed other structures on the same substrate but, in some aspects, even surpassed the corresponding structures on ITO–glass substrates. At the 10^−3^ M concentration (Figure 9a, red line), the SERS signal intensity produced by Ag-ZnO on ITO-PET was comparable to or even slightly higher than that of the corresponding structure on ITO–glass, with sharper peak shapes and lower background noise. In particular, the aromatic ring stretching vibration peaks at 1364 cm^−1^ and 1510 cm^−1^ showed an extremely strong enhancement effect. Even at low concentrations, especially 10^−5^ M (Figure 9c, red line), Ag-ZnO on ITO-PET still generated exceptionally clear SERS signals, with all major characteristic peaks clearly discernible. The signal intensity was significantly higher than those of all the other substrates, including the corresponding structure on ITO–glass. This indicates that the Ag-ZnO composite structure on flexible substrates may possess a unique enhancement mechanism, making it particularly suitable for detecting low-concentration molecules.

Systematic comparison of SERS data from ITO–glass (Figure 8) and ITO-PET (Figure 9) substrates revealed distinct substrate-dependent performance characteristics. Flexible ITO-PET substrates generally enhanced the performance of Ag-ZnO and pristine ZnO NRs while diminishing the relative advantages of Au-ZnO structures. SERS substrates on flexible substrates, particularly Ag-ZnO composites, demonstrated superior low-concentration detection capabilities (10^−5^ M) with improved signal retention and enhanced peak clarity compared to rigid substrates. The SERS spectra on flexible substrates exhibited altered peak shapes and intensity ratios, with the C=C stretching vibration at 1652 cm^−1^ showing consistently stronger relative intensities. These performance variations arise from multiple interconnected factors. The flexible PET substrate may experience subtle deformation during measurement, inducing piezoelectric polarization in ZnO NRs. Minimal stresses may possibly generate significant electric fields that enhance SERS through amplification of surface plasmon resonance effects, electrostatic concentration of target molecules at active sites, and modification of metal–ZnO interfacial band structures that promote charge transfer processes. The structural characteristics observed in SEM and XRD analyses contribute significantly to performance differences, with the multi-directional ZnO nanorod orientations on PET substrates creating diverse molecular adsorption environments and generating complex strain distributions under deformation. The unique metal–ZnO interfacial architectures on flexible substrates, including Ag film bridging structures, facilitate enhanced coupling between piezoelectric fields and plasmonic resonances during substrate deformation. These interfacial modifications, combined with the altered crystallographic orientations and morphological characteristics on PET substrates, create synergistic enhancement mechanisms that extend beyond simple additive effects. The resulting performance improvements establish flexible substrates as advantageous platforms for SERS applications requiring high sensitivity and dynamic response capabilities.

### 3.9. Detection Performance of Metal–ZnO Nanocomposites on Rigid Substrates

**Figure 10:**
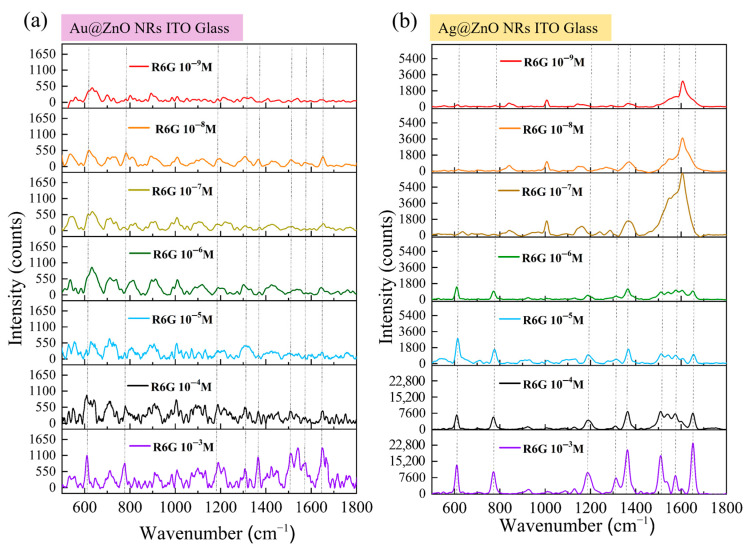
SERS performance for (**a**) Au-ZnO and (**b**) Ag-ZnO NCPs on ITO–glass substrates detecting R6G with 10^−3^ M to 10^−9^ M concentrations.

To thoroughly evaluate the detection performance of the prepared SERS substrates, we systematically tested the ability of both Au-ZnO and Ag-ZnO composite structures on ITO–glass substrates to detect R6G molecules at extremely low concentrations. Figure 10 illustrates the detection performance of the two SERS substrates in the concentration range of 10^−3^ M to 10^−9^ M. Figure 10a shows the SERS response of Au-ZnO NCPs, prepared by the PLIP method with varying concentrations of R6G. As the R6G concentration decreased, the SERS signal intensity gradually diminished. At higher concentrations (10^−3^ M and 10^−4^ M, purple and black lines in Figure 10a), all characteristic peaks of R6G were clearly visible, with a high signal intensity and clear peak shapes. This aligns with the uniform distribution of Au nanoparticles observed in previous SEM analyses (Figure 4a,b). As shown in SEM, Au nanoparticles formed a uniformly distributed coating on the ZnO nanorod surfaces, with particle sizes of approximately 10–20 nm. This morphology is conducive to forming high-density “hot spot” regions, generating strong SERS signals. When the concentration dropped to 10^−5^ M and 10^−6^ M (blue and green lines), the main characteristic peaks were still discernible. However, the signal intensity was noticeably weaker, and the signal-to-noise ratio (SNR) decreased. As the concentration further decreased to 10^−7^ M and 10^−8^ M (yellow and orange lines), the main characteristic peaks of R6G were still identifiable but became more blurred, with a further reduction in the SNR. At the extremely low concentration of 10^−9^ M (red line), most characteristic peaks were difficult to clearly distinguish from the background noise; only the peak at 612 cm^−1^ was barely discernible. This indicates that the detection limit of the Au-ZnO composite structure is approximately between 10^−8^ M and 10^−9^ M, which is already quite excellent detection sensitivity.

Figure 10b shows the SERS response of Ag-ZnO NCPs, prepared by thermal evaporation with varying concentrations of R6G. Compared to Au-ZnO, Ag-ZnO demonstrated a significantly stronger SERS enhancement effect and a lower detection limit. In the high concentration range (10^−3^ M to 10^−5^ M; purple, black, and blue lines), all R6G characteristic peaks were exceptionally clear, with a high signal intensity, sharp peak shapes, and excellent SNRs. Notably, at the 10^−4^ M concentration, the signal intensity produced by Ag-ZnO was several times higher than that of Au-ZnO at the same concentration, showcasing the significant advantage of the Ag structure. This performance difference is directly related to the distinct structural features observed in SEM (Figure 5b). From the surface morphology observation, the Ag covered the top and upper sidewalls of the ZnO NRs in the form of a continuous thin film, forming a smooth and uniform metal layer, rather than dispersed nanoparticles. This continuous film structure may provide a more stable and powerful surface plasmon resonance effect. When the concentration decreased to 10^−6^ M and 10^−7^ M (green and brown lines), Ag-ZnO could still provide remarkably clear SERS spectra, with all major characteristic peaks clearly identifiable and only a slight decrease in signal intensity. According to the XRD results (blue line in Figure 7a), Ag-modified ZnO NRs not only retained their original crystal structure but also introduced a distinct Ag crystalline phase, indicating the formation of a well-crystallized Ag film. This highly crystalline Ag coating may generate more stable and stronger surface plasmon resonance modes, beneficial for low-concentration detection. At even lower concentrations of 10^−8^ M and 10^−9^ M (orange and red lines), Ag-ZnO still exhibited astonishing detection capability. At 10^−8^ M, all major characteristic peaks were clearly visible, and at the extremely low concentration of 10^−9^ M, multiple peaks could still be reliably identified. This indicates that the detection limit of the Ag-ZnO composite structure can reach 10^−9^ M or even lower, demonstrating extremely high SERS sensitivity. Even though the LSPR peak of Ag (approximately 420 nm, as shown in the inset of Figure 6c) has a certain mismatch with the 633 nm excitation wavelength, Ag-ZnO still provided extremely strong SERS enhancement, further proving the excellent optical properties and potential chemical enhancement mechanisms of the Ag thin film coating. Based on the SERS data in Figure 10, and combined with the microstructural characterization results, we can calculate the EFs for both composite structures. Using the characteristic peak at 1364 cm^−1^ for calculation and referencing the R6G signal at 10^−3^ M on pristine ITO–glass, rough estimations yielded a maximum EF of approximately 10^5^−10^6^ for Au-ZnO, while the maximum EF for Ag-ZnO reached 10^7^−10^8^.

### 3.10. Detection Performance of Metal–ZnO Composite Structures on Flexible Substrates

**Figure 11:**
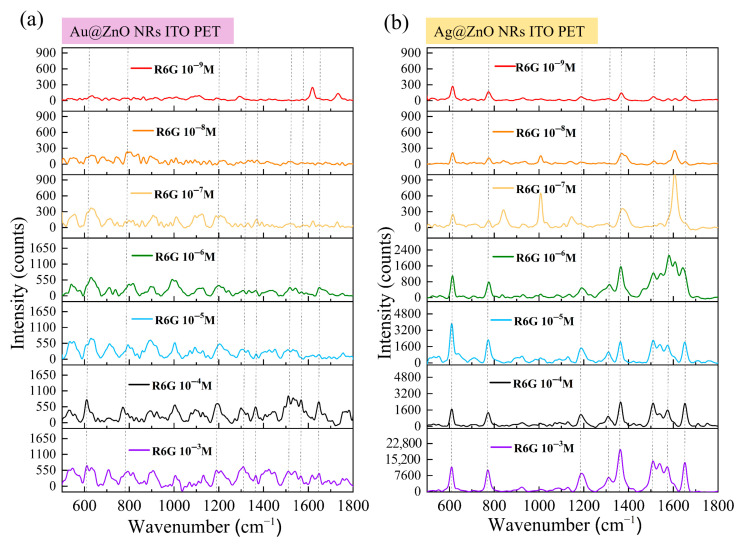
SERS performance for (**a**) Au-ZnO and (**b**) Ag-ZnO NCPs on ITO-PET substrates detecting R6G with 10^−3^ M to 10^−9^ M concentrations.

Figure 11a shows the SERS response of Au-ZnO composite structures, prepared by the PLIP method, to varying concentrations of R6G on flexible ITO-PET substrates. Compared to the ITO–glass substrate, the trend of decreasing SERS signal intensity with lower R6G concentration was similar, but distinct characteristics were observed under certain concentration conditions. At higher concentrations (10^−3^ M and 10^−4^ M), all characteristic R6G peaks were clearly visible in the SERS spectra from Au-ZnO on ITO-PET. However, the peak shapes were slightly different compared to the corresponding structures on ITO–glass and the relative intensities of certain vibrational modes, such as 774 cm^−1^ and 1186 cm^−1^. These spectral variations correlate directly with substrate-dependent structural differences revealed in earlier characterizations. SEM analysis demonstrated that Au nanoparticles on PET substrates formed clustered and networked arrangements rather than uniform distributions, creating distinct hot spot patterns and electromagnetic enhancement modes that modify SERS spectral characteristics. The different surface chemical properties of PET substrates compared to glass influence R6G molecular adsorption behavior and distribution patterns, further affecting signal characteristics. Additionally, the altered ZnO nanorod architecture on PET substrates, characterized by diverse orientations and higher densities, as observed in XRD and SEM analyses, provides modified deposition environments for Au nanoparticles that ultimately influence SERS performance. At lower concentrations (10^−5^ M to 10^−7^ M), the Au-ZnO detection performance on ITO-PET remained comparable to that of glass-supported structures, maintaining detection limits between approximately 10^−8^ M and 10^−9^ M.

Figure 11b shows different behavior for Ag-ZnO NCPs on flexible ITO-PET substrates, which exhibited superior SERS performance compared to ITO–glass substrates. The enhancement was most pronounced in the medium-to-low concentration range (10^−6^ M to 10^−8^ M), correlating with several substrate-dependent structural modifications. SEM analysis revealed that Ag films on PET substrates formed complex three-dimensional networks and bridging connections that create more numerous and effective hot spot regions compared to simpler coverage patterns observed on glass substrates. UV-Vis spectroscopy demonstrated stronger surface plasmon resonance absorption characteristics for Ag-ZnO on PET substrates, directly contributing to improved SERS capabilities. XRD analysis showed distinct differences in ZnO crystal characteristics following Ag modification on PET versus glass substrates, suggesting altered interfacial structures that promote more favorable charge transfer conditions. These substrate-dependent performance variations establish that the combination of flexible PET substrates with specific metal–ZnO architectures produces synergistic effects that enhance SERS capabilities beyond simple material property additions, particularly for Ag-based composite systems.

Detection performance evaluation based on Figure 11 data revealed substrate-dependent variations that differed between metal types. Au-ZnO maintained similar detection limits (10^−8^ M) on both substrate types, while Ag-ZnO achieved improved performance on ITO-PET (10^−9^ M detection limit) compared to ITO–glass substrates. Enhancement factor calculations yielded maximum EF values of 10^6^–10^7^ for Au-ZnO and 10^8^–10^9^ for Ag-ZnO on flexible substrates. These results demonstrate that substrate material selection significantly influences SERS performance, with the extent of influence varying according to metal type. Ag-ZnO systems exhibited greater substrate sensitivity, showing markedly superior performance on flexible PET substrates, whereas Au-ZnO performance remained relatively consistent across different substrate materials, indicating distinct substrate–metal interaction mechanisms that govern enhancement efficiency.

### 3.11. Piezoelectric-Enhanced SERS Effect Induced by Bending Flexible Substrates

**Figure 12:**
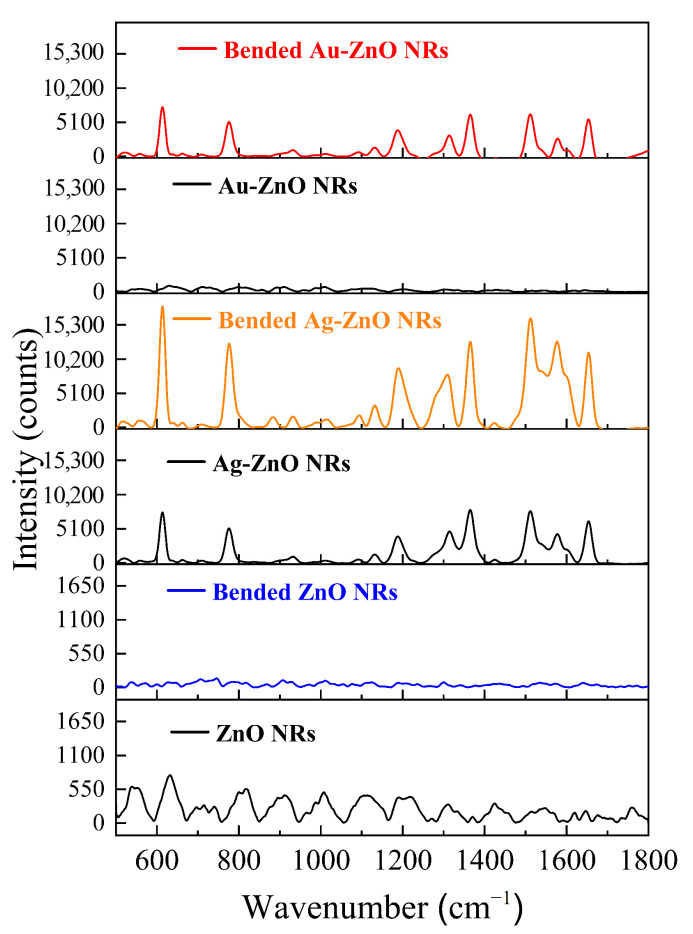
Piezoelectric-enhanced SERS effect of ZnO NRs NRs and metal–ZnO NCPs on ITO-PET flexible substrates in the normal and bent state detecting 10^−5^ M R6G.

To directly confirm the contribution of the piezoelectric effect to SERS enhancement, we performed bending tests on metal–ZnO composite structures on ITO-PET flexible substrates. We compared the SERS performance of the same sample in bent and unbent states. Figure 12 shows a comparison of the SERS spectra for ZnO NRs and for Au-ZnO and Ag-ZnO NCPs detecting 10^−5^ M R6G, in both bent and unbent states. The upper part of Figure 12 (red line) shows the SERS response of the Au-ZnO/PET to 10^−5^ M R6G after the PET substrate was bent. Compared to the unbent state (black line), the SERS signal intensity after substrate bending was significantly enhanced. In the unbent state, clear R6G characteristic peaks were barely detectable on the Au-ZnO/PET substrate; however, when the substrate was bent, all R6G characteristic peaks immediately became clearly visible. The signal intensity increased by tens to even hundreds of times. This enhancement directly confirms piezoelectric effect contributions beyond simple morphological changes from substrate deformation. When the flexible PET substrate bends, ZnO NRs experience strain-induced piezoelectric polarization, creating localized electric fields that enhance SERS through multiple mechanisms. The piezoelectric field increases the R6G molecular concentration at active sites through electrostatic adsorption, which is particularly effective for low-concentration samples where conventional enhancement mechanisms become limited. The diverse ZnO nanorod orientations on PET substrates, as observed in SEM analysis, generate complex strain distributions under bending that produce heterogeneous polarization patterns optimized for molecular capture. Additionally, the piezoelectric field couples with Au nanoparticle surface plasmon resonance to amplify localized electromagnetic fields, while the networked Au nanoparticle architecture on PET substrates facilitates this coupling through enhanced field interactions. The piezoelectric field also modifies Au-ZnO interfacial band structures, promoting charge transfer processes that augment chemical enhancement mechanisms. XRD analysis revealed specific crystal orientation changes in Au-modified ZnO NRs on PET substrates, suggesting that these interfacial modifications create favorable conditions for piezoelectric-enhanced charge transfer during substrate deformation.

Figure 12 also shows the change in SERS performance of the Ag-ZnO/PET before and after bending. Unlike the Au-ZnO system, Ag-ZnO already exhibited a considerably strong SERS signal in the unbent state (black line). All R6G characteristic peaks were clearly visible, with a high signal intensity and sharp peak shapes. This is consistent with the previously observed superior basic SERS performance of Ag-ZnO compared to Au-ZnO. However, when the substrate was bent (orange line), the SERS signal intensity of Ag-ZnO was still significantly enhanced, with an increase of approximately two to three times. Although this enhancement was not as dramatic as in the Au-ZnO system, considering the already high performance of Ag-ZnO in the unbent state, this enhancement is still very significant, further confirming the enhancing contribution of the piezoelectric effect to the SERS signal. This enhancement directly confirms piezoelectric effect contributions beyond simple morphological changes from substrate deformation. When the flexible PET substrate bends, ZnO NRs experience strain-induced piezoelectric polarization, creating localized electric fields that enhance SERS through multiple mechanisms. The piezoelectric field increases the R6G molecular concentration at active sites through electrostatic adsorption, which is particularly effective for low-concentration samples where conventional enhancement mechanisms become limited. The diverse ZnO nanorod orientations on PET substrates, as observed in SEM analysis, generate complex strain distributions under bending that produce heterogeneous polarization patterns optimized for molecular capture. Additionally, the piezoelectric field couples with Au nanoparticle surface plasmon resonance to amplify localized electromagnetic fields, while the networked Au nanoparticle architecture on PET substrates facilitates this coupling through enhanced field interactions. The piezoelectric field also modifies Au-ZnO interfacial band structures, promoting charge transfer processes that augment chemical enhancement mechanisms. XRD analysis revealed specific crystal orientation changes in Au-modified ZnO NRs on PET substrates, suggesting that these interfacial modifications create favorable conditions for piezoelectric-enhanced charge transfer during substrate deformation.

It should be noted that this mechanistic interpretation is based on indirect evidence from our systematic structural and performance characterizations. Future studies incorporating direct piezoelectric field measurements would provide valuable validation of the proposed enhancement pathways. While direct measurement of piezoelectric fields was not performed in this study, the observed enhancement can be understood through established piezoelectric theory. When ZnO nanorods experience mechanical strain during substrate bending, the asymmetric wurtzite crystal structure generates internal electric fields due to charge separation. The diverse nanorod orientations on PET substrates (as observed in XRD analysis, Figure 7b) create complex strain distributions that produce heterogeneous polarization patterns optimized for molecular capture. The piezoelectric fields likely enhance SERS through electrostatic concentration of R6G molecules at active sites, modification of Au-ZnO interfacial band structures that promote charge transfer, and coupling with Au nanoparticle surface plasmon resonance to amplify electromagnetic fields.

Table 1 provides a comprehensive summary of the SERS enhancement factors obtained for all substrate configurations at the 10^−5^ M R6G concentration. The data clearly demonstrate that the flexible ITO-PET substrates generally outperformed the rigid ITO–glass substrates across all configurations. While Ag-ZnO composites exhibit the highest baseline SERS activity, Au-ZnO systems show the most dramatic piezoelectric enhancement upon substrate bending, with signal amplification reaching 50–100 times. All metal–ZnO composites benefit from piezoelectric effects, though the magnitude varies significantly between different metal types and substrate configurations. These results establish the critical importance of substrate selection and mechanical responsiveness in optimizing SERS performance for flexible sensor applications.

## 4. Conclusions

This study systematically investigated how substrate flexibility and metal deposition methods influence piezoelectric-enhanced SERS performance in ZnO nanorod composites. ZnO NRs on rigid ITO–glass substrates exhibited strong c-axis orientation and larger diameters (80–100 nm), whereas those on flexible ITO-PET substrates demonstrated smaller diameters (60–80 nm), a higher density, and diverse orientations that enhanced piezoelectric responsiveness. The metal deposition methods produced fundamentally different architectures: laser-induced photolysis created uniformly distributed Au nanoparticles with comprehensive surface coverage, while thermal evaporation formed continuous Ag films concentrated on nanorod upper surfaces. SERS performance measurements revealed that flexible substrates generally outperformed rigid counterparts, particularly at low concentrations. Ag-ZnO/PET achieved detection limits of 10^−9^ M with enhancement factors of 10^8^–10^9^, while Au-ZnO/PET reached 10^−8^ M detection limits. Substrate bending experiments provided direct evidence for piezoelectric enhancement, with Au-ZnO/PET exhibiting 50–100-fold signal amplification and Ag-ZnO/PET showing 2–3-fold enhancement upon mechanical deformation. These differential responses reflect distinct coupling mechanisms between piezoelectric fields and metal nanostructures. The findings establish key design principles for piezoelectric-enhanced SERS sensors: while Ag-ZnO composites provide superior baseline SERS activity, Au-ZnO structures demonstrate greater piezoelectric responsiveness. Optimal sensor design requires balancing intrinsic enhancement capabilities with mechanical responsiveness according to application requirements. This work establishes fundamental structure–property relationships in piezoelectric-enhanced SERS and provides practical guidelines for developing high-performance flexible sensors for biosensing, environmental monitoring, and point-of-care diagnostics.

## Figures and Tables

**Table 1:** Summary of SERS enhancement factors and piezoelectric enhancement effects for metal–ZnO nanocomposites at the 10^−5^ M R6G concentration using the 1364 cm^−1^ peak as a reference.

Substrate Type	Flat State EF	Bent State EF	Enhancement Factor *	Detection Quality
Rigid Substrates (ITO–glass)				
ZnO NRs	~10^2^	N/A	N/A	Weak signal
Au-ZnO NCPs	~10^4^	N/A	N/A	Clear peaks
Ag-ZnO NCPs	~10^5^	N/A	N/A	Strong signal
Flexible Substrates (ITO-PET)				
ZnO NRs	~10^3^	~10^4^	~10×	Improved signal
Au-ZnO NCPs	~10^4^	~10^6^	50–100×	Dramatic increase
Ag-ZnO NCPs	~10^6^	~10^6.5^	2–3×	Further enhanced

* Enhancement Factor = (Bent State EF)/(Flat State EF).

## Data Availability

The original contributions presented in this study are included in the article. Further inquiries can be directed to the corresponding authors.
